# Inter–laboratory precision and relative accuracy of mechanised and nonmechanised systems for blood gas analysis

**DOI:** 10.1155/S1463924680000302

**Published:** 1980

**Authors:** Alain Feuillu, Michel Catheline, Andre Le Treut

**Affiliations:** Laboratory of the Intensive Therapy Unit Pontchaillou Hospital Rue Henri le Guillou Rennes 35033 France


### Inter-laboratory precision and relative accuracy of mechanised and non-mechanised systems for blood gas analysis

**Alain Feuillu, Michel Catheline and Andre Le Treut**
*Laboratory of the Intensive Therapy Unit, Pontchaillou Hospital, Rue Henri le Guillou, 35033 Rennes, France*

### Introduction

In recent years, quality control has been extended to an ever-increasing number of biochemical parameters. Its application to blood-gas analysis is complicated by two main factors: the need for reliable, reproducible quality control materials and the variability of calibration standards among the different clinical departments using blood gas apparatus [1]. Moreover, automatic analysers are becoming increasingly sophisticated and complex, which may simplify handling but inevitably increases the risk of error.

The purpose of the present study was to compare automatic and non automatic systems for precision and accuracy on the strength of the results of an inter-laboratory quality control survey.

### Materials and methods

Twenty-two hospital laboratories from all over France participated in the study, which involved seven mechanised and 15 manual analysers. The two systems were considered separately in order to investigate the possible effects on precision of differences in calibration and operation. All the mechanised instruments were of the same type (ABL I, ABL II from Radiometer). The manual instruments comprised eight Radiometer BMS, two Corning 165 and five Instrumentation Laboratory 413 analysers. All the instruments were in routine use in specialised or non-specialised hospital clinical chemistry laboratories.

A quality control product at three levels (acidosis, normal, alkalosis) was studied. The Precebéo stated values for these three levels are shown in Table 1 and were calculated from multiple measurements with different blood-gas analysers. A single batch of the product was used for all the laboratories participating in the survey. The differences between the three levels must be great enough to enable measurement errors to be characteristic. The quality control programme was conducted over an 18-month period with each laboratory performing ten measurements a month for each level, and for the three parameters measured (pH, $pCO_2$ and $pO_2$), so giving a total of 90 analyses per month for each laboratory. At the end of each month, the data from these 22 laboratories were submitted to quality control assessment, with emphasis on two criteria (for each of the three parameters and three levels): that is precision and accuracy.

**Table 1. Manufacturer's stated values for quality control solutions**

| | Acidosis | Normal | Alkalosis |
|---|---|---|---|
| pH | 7.09 ± 0.02 | 7.41 ± 0.02 | 7.60 ± 0.02 |
| $pCO_2$ (kPa) | 2.39 ± 0.27 | 5.19 ± 0.40 | 7.18 ± 0.66 |
| $pO_2$ (kPa) | 19.41 ± 1.33 | 13.03 ± 0.66 | 7.58 ± 0.66 |

### Precision

Inter-laboratory precision was determined by variance analysis using the Fisher test reference. The monthly variance for each laboratory was compared with that for all participating laboratories. When the variance for a laboratory was less than or not significantly different to the mean variance, according to the Fisher table, the result was considered 'precise'. Conversely, if the above condition was not met, the result was considered 'imprecise'.

### Accuracy

Accuracy was determined by comparing the monthly mean for a laboratory with the overall mean, having excluded outliers of more than two S.D.S. The overall mean was taken as the target value, in accordance with statistical computations used in most quality control surveys. When the monthly mean for a laboratory did not differ significantly from the overall mean as defined above, the result was considered 'accurate'. The results were judged according to the 't' test for standard deviation of the random error exceeding obvious wrong results and at the 5% level. Conversely, if this condition was not met, the result was considered 'inaccurate'.

### Results

For each parameter, the results from laboratories using mechanised equipment were considered separately from those of laboratories using manual equipment. The percentages of accurate and precise results were compared for each of these two groups. These results are shown Table 2 and Figure 1.

### pH

For both acidosis and normal levels, the mechanised analysers gave a significantly higher percentage of accurate results than did the manual apparatus. However, for alkalosis results, the difference disappeared. For both types of apparatus precision was similar (Tables 2 and 3).

### $pCO_2$

With the exception of the acidosis levels, accuracy was much greater for the manual equipment. Precision, however, was better with the mechanised apparatus for acidosis and alkalosis. In general, the distribution of the results was closer for the mechanised than for the manual apparatus. The results are confirmed by reference to Tables 2 and 3.

### $pO_2$

The manual apparatus showed a greater percentage of accurate results than did the mechanised apparatus for the three levels, from the lowest to the highest values. The same was true of precision, which was greater for the manual systems, as shown by the distribution of results with Tables 2 and 3.

**Table 2. Percentages of "precise" and "accurate" results obtained with mechanised and manual equipment for measurement of pH, $pCO_2$ and $pO_2$.**
**NS = Results not significantly different.**

| | | PRECISE | | | | ACCURATE | | | |
|---|---|---|---|---|---|---|---|---|---|
| | | Mechanised | Manual | Difference | p | Mechanised | Manual | Difference | p |
| pH | Acidosis | 89.5 % | 85.7 % | 3.8 | NS | 68.1 % | 43.3 % | 24.8 | 0.05 |
| | Normal | 85.2 % | 84.1 % | 1.1 | NS | 86.3 % | 51.5 % | 34.8 | 0.05 |
| | Alkalosis | 89.5 % | 88.4 % | 1.1 | NS | 63.6 % | 65.5 % | 1.9 | NS |
| $pCO_2$ | Acidosis | 95.0 % | 92.1 % | 2.9 | NS | 59.0 % | 64.5 % | 5.5 | NS |
| | Normal | 77.7 % | 88.6 % | 10.9 | NS | 22.7 % | 41.0 % | 18.3 | 0.05 |
| | Alkalosis | 97.4 % | 84.7 % | 12.7 | NS | 18.1 % | 56.9 % | 38.8 | 0.01 |
| $pO_2$ | Acidosis | 85.7 % | 92.2 % | 6.5 | NS | 45.4 % | 61.7 % | 16.3 | NS |
| | Normal | 76.2 % | 92.1 % | 15.9 | 0.05 | 31.5 % | 65.4 % | 33.9 | 0.01 |
| | Alkalosis | 63.1 % | 91.5 % | 28.4 | 0.01 | 21.4 % | 52.1 % | 30.7 | 0.01 |

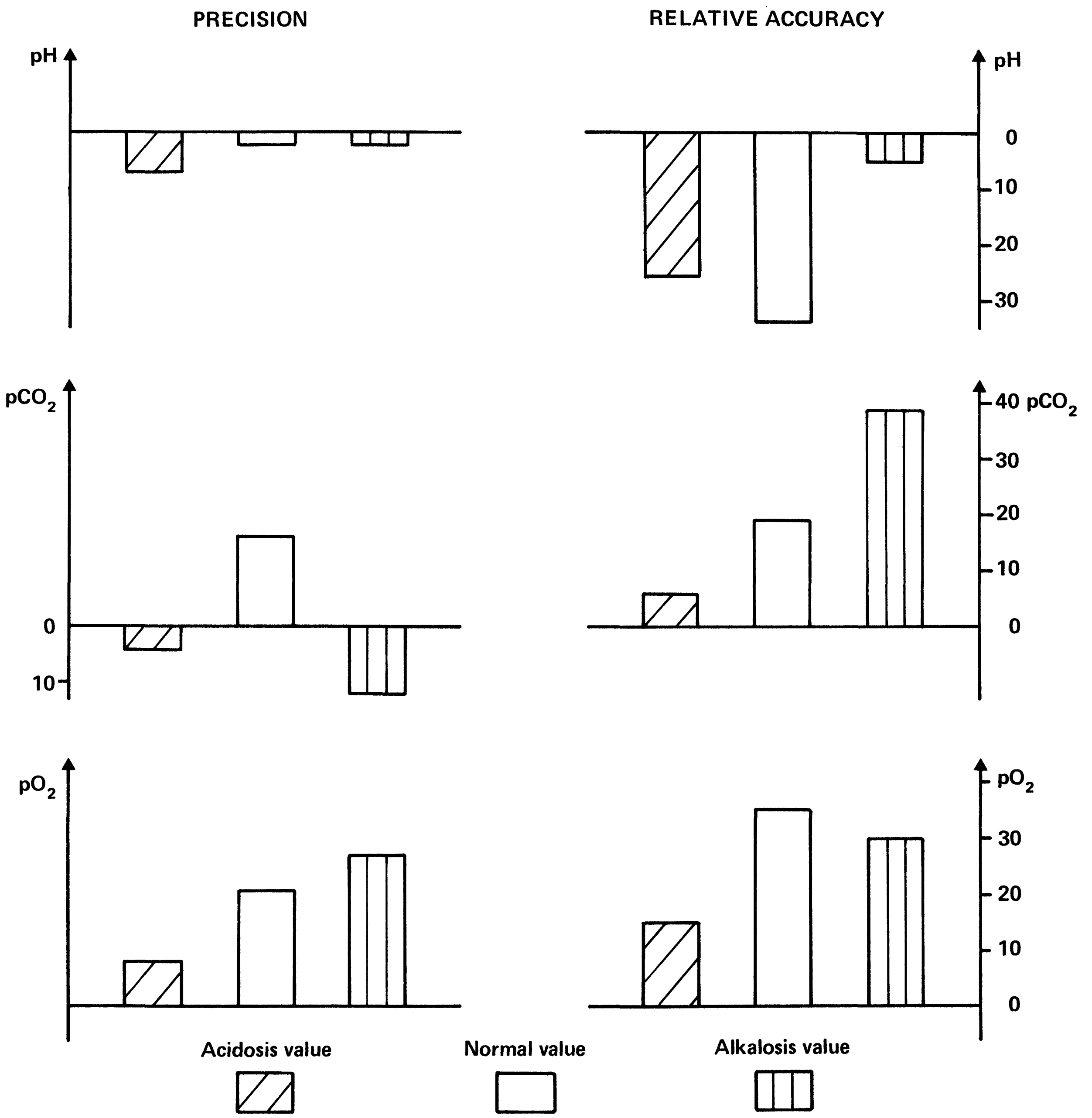

*Figure 1. Differences of 'precise' results (left) or 'accurate' results (right) between mechanised and manual systems. (Differences favourable to manual equipment are plotted above the abscissa line and vice versa).*

### Discussion

Sources of error revealed during surveys of this type are very varied. Apart from poor sample handling during the analysis, some are common to all analytes studied and some are specific to one only. The lack of a mechanised system for introducing solutions into the different analysers is a possible source of contamination. The calibrating gases must be carefully measured and humidified to ensure correct calibration. The electrodes themselves or the electrode membranes may be faulty. Errors resulting from these defects can cause results for one of the laboratories to be outside the allowable limits of error for one or more of the analytes.

The manual apparatus was less accurate than the mechanised apparatus for pH measurement. The degree of precision calculated by variance analysis is about the same, whatever the type of apparatus used. This is shown on Table 2 and illustrated on Figure 1. This suggests that the poorer accuracy shown by the manual equipment is not related to poor replicability of the measurements but rather to the manual method of calibration. The single-point calibration used by many manual operators is inadequate. Regular two-point calibration as used in the mechanised apparatus, with a correction for drift, may account for the greater accuracy found with the systems.

By contrast, $pCO_2$ measurements were less accurate with the mechanised systems. Whereas the degree of replicability is about the same for both types of equipment (Table 2) with no loss of accuracy whichever the type of apparatus is used when blood $pCO_2$ is low (Figure 1). Results obtained with the mechanised systems are inaccurate, especially for normal and low $pCO_2$ values. For the type of apparatus used in this survey preanalysed mixtures of gases were not used. Carbon dioxide and room air mixed in a gas-mixing apparatus are used. With the manual systems, gas mixtures precalibrated with carbon dioxide at two partial pressures are always used. This difference in calibration procedure may account for the lower degree of accuracy found with the mechanised as compared with the manual systems.

$pO_2$ measurement was also less accurate with the mechanised apparatus. The difference could be attributed to the fact that the mechanised systems use an electrical zero wheras the manual apparatus is calibrated for $pO_2$ with two gas mixtures, nitrogen-carbon dioxide for zero, and oxygen-carbon dioxide for $pO_2$ values of about 13.30 kPa. Nevertheless, the data in Table 2 and Figure 1 show clearly that the mechanised equipment is less precise than the manual equipment. Moreover, there was good correlation between the percentage of accurate results and the percentage of precise results (Figure 2, $v = 0.95, p < 0.01$). Thus the poor accuracy of the mechanised systems was consistent with the fall in replicability first seen for values near the normal and becoming pronounced with elevated $pO_2$ values. Poor replicability, however, cannot be attributed wholly to handling errors. The range between the extreme values (Table 3) reflects the influence of outlying results. No correlation was found for $pO_2$, for $pCO_2$ or even for pH measurements, with the percentage of precise results

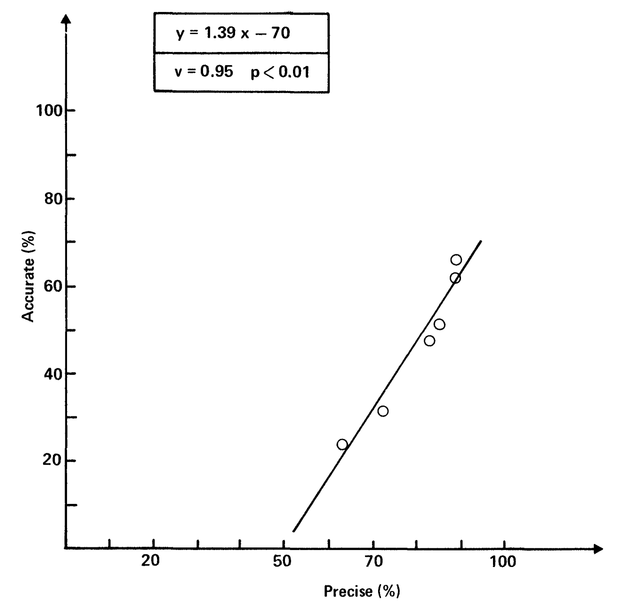

*Figure 2. Relationship between percentages of precise (x) and accurate (y) $pO_2$ measurements.*

($v = 0.53$; not significant). So much so that apart from calibration-related inaccuracies, the mechanised systems appear to involve a certain degree of contamination which does not produce an increase in variance and a consequent fall in accuracy. One possible source of contamination could be related to the way in which samples are introduced into the analysers. Inadequately gas-tight measuring chambers might also introduce contamination.

Clearly, the ideal material for quality control of blood gas analysis should be composition and properties identical to those of blood samples being tested. A buffer solution, even if very complex in composition, differs from whole blood and is less satisfactory as a control material. Thus the response of the glass electrode and the residual liquid junction potential are different for buffers and for whole blood, but since no sufficiently stable commercial whole blood preparation is available, aqueous solutions provide one simple means of assessing the performance of laboratories not specialising in blood gas analysis.

### Conclusion

The comparative data obtained in this inter-laboratory survey of mechanised and non-mechanised blood-gas measurement systems point to the need for quality control at three levels, and highlight the fact that inaccuracies often occur

**Table 3. Range of values obtained with mechanised and manual equipment for measurement of pH, $pCO_2$ and $pO_2$**
**$\Delta$ = Absolute value of the difference between the extremes.**

| | | Mechanised | | Manual | |
|---|---|---|---|---|---|
| pH | Acidosis | 7.020 – 7.164 | $\Delta = 0.144$ | 7.040 – 7.160 | $\Delta = 0.120$ |
| | Normal | 7.341 – 7.464 | $\Delta = 0.123$ | 7.340 – 7.540 | $\Delta = 0.200$ |
| | Alkalosis | 7.539 – 7.654 | $\Delta = 0.115$ | 7.540 – 7.620 | $\Delta = 0.080$ |
| $pCO_2$ (kPa) | Acidosis | 1.80 – 2.66 | $\Delta = 0.86$ | 1.86 – 3.06 | $\Delta = 1.20$ |
| | Normal | 4.12 – 5.85 | $\Delta = 1.73$ | 4.26 – 5.19 | $\Delta = 0.93$ |
| | Alkalosis | 6.38 – 7.45 | $\Delta = 1.07$ | 5.72 – 7.75 | $\Delta = 2.03$ |
| $pO_2$ (kPa) | High | 15.29 – 20.74 | $\Delta = 5.45$ | 17.02 – 18.89 | $\Delta = 1.81$ |
| | Normal | 11.17 – 15.83 | $\Delta = 4.66$ | 12.37 – 16.36 | $\Delta = 3.99$ |
| | Low | 5.45 – 11.44 | $\Delta = 5.99$ | 5.32 – 8.25 | $\Delta = 2.93$ |

with extreme values (at low $pCO_2$ and high $pO_2$). The findings also suggest that fully mechanised systems are neither more accurate nor more precise under all circumstances. Mechanised equipment lacked replicability in $pO_2$ measurements and its use seemed to be associated with a certain degree of contamination.

An important conclusion of this study is that two-point calibration is to be preferred for all measurements, and that equipment involving single-point calibration (non-mechanised) for pH measurement, mechanised for measurements of $pCO_2$ and $pO_2$) is inadequate.

Undoubtedly, tonometry is still the best method of controlling blood gas analysis, but it is beyond the reach of many laboratories. For this reason, the results of the present survey have important fractional implications.

REFERENCES

[1] Minty, B.D. and Nunn, J.F., *Annals of Clinical Biochemistry*, 1977, **14**, 245.

[2] Evans, J.R., *Annals of Clinical Biochemistry*, 1978, **15**, 168.

[3] Komjathy, Z.L., Mathies, J.C., Parker, J.A. and Schreiber, H.A., *Clinical Chemistry*, 1976, **22**, 1399.

[4] Maas. A.H., Veefkind, A.H., Van-den-Camp, R.A., Teunissen, A.J. and Winckers, E.K., *Clinical Chemistry*, 1977, **23**, 1718.

[5] Noonan D.C. and Komjathy Z.L., *Clinical Chemistry*, 1976, **22**, 1818.

[6] Schwartz, O., Methodes statistiques a l'usage des Medecins et des Biologistes, Editions Flammarion II, 1969, 173.

[7] Ksorensen, Radiometer A.J. Copenhagen, Denmark Quality control and standards National Bureau of Standards, Special Publication 450, Blood pH gases and electrolytes U.S. Dept of Commerce, Washington D.C. June 1977 285.

### Erratum

*The Journal of Automatic Chemistry*, October 1979, 1, 5, 273-281.

### An evaluation of the Kodak Ektachem system for the determination of glucose and urea

**R. Haeckel, O. Sonntag and K. Pethy.**

The authors have asked that the following errors in the above paper be brought to the attention of readers.

On page 276 in the second paragraph under the heading "Accuracy" reference is twice made to "Figures 4 and 7". In both cases this should read "Figures 4 to 7".

In Figures 11 and 12 on page 280, the line and reference [14] should have been deleted.

In Table 10 the methotrexate concentration should have been given in μmol.

Some additional information has been provided for Table 9 and for completeness, we have reproduced the entire table.

**Table 9 Refound values of glucose and urea in pool sera to which various components were added. In the absence of exogenic compounds mean values (x) and standard deviations (s) were calculated from 15 determinations. Results of analysis which are outside the range of confidence (x ± 3s), are marked with an asterisk. The range of confidence is for glucose 11.14 – 14.08 mmol/1 and for urea 6.23 – 6.68 mmol/1.**

| Trade name | I.N.N. (1) | concentration mg/l | glucose (mmol/l) | urea (mmol/l) |
|---|---|---|---|---|
| Amuno | indometacinum | 4 | 13.28 | 6.47 |
| Butazolidin | phenylbutazonum | 12 | 13.06 | 6.51 |
| Metalcaptase | D-penicillaminum | 36 | 12.35 | 6.53 |
| Prolixan | azopropazon-d ihydrat | 36 | 13.45 | 6.57 |
| Resochin | chloroquinum | 5 | 12.70 | 6.57 |
| Tanderil | oxyphenbutazonum | 12 | 12.67 | 6.46 |
| Aponal | doxepinum | 6 | 12.76 | 6.43 |
| Megaphen | phenothiazinum | 20 | 13.11 | 6.55 |
| Multum | chlordiazeposidum | 1.8 | 12.26 | 6.56 |
| Aspirin | acidum acetylosali-cylicum | 100 | 13.59 | 6.45 |
| Dolviran | acidum acetylocali-cylicum, etc. | – | – | – |
| Novalein | novaminsulfonum | 80 | 12.79 | 6.45 |
| Benemid | probenecidum | 40 | 13.36 | 6.42 |
| Uriovac | benzbromaronum | 8 | 13.17 | 6.44 |
| Zyloric | allopurinolum | 18 | 12.71 | 6.39 |
| Anglografin | acidum trijodbenzoicum | 1300 | 13.07 | 6.55 |
| Biligrafin | adipinyltrijodanilidum | 200 | 12.20 | 6.39 |
| Urografin | acidum trijodbenzoicum | – | – | – |
| Binotal 500 | aminobenzylpenicillinum | 180 | 13.23 | 6.46 |
| Hostacyclin | tetracyclinum | 40 | 16.99* | 6.77* |
| Paraxin | chloramphenicolum | 600 | 16.01* | 6.64 |
| Buscopan | hyoscin-N-butylbrominum | 2 | 13.16 | 6.59 |
| Cebion | acidum ascorbicum | 80 | 13.24 | 6.42 |
| Polybion | Vitamin B complex | 2.3 | 12.57 | 6.39 |

*Table continues overleaf*

